# Troponin I Cutoff for Non-ST-Segment Elevation Myocardial Infarction in Sepsis

**DOI:** 10.1155/2022/5331474

**Published:** 2022-05-27

**Authors:** Meng-Ko Tsai, Chao-Hung Lai, Chia-Lien Hung, Keng-Yi Wu

**Affiliations:** ^1^Division of Rheumatology, Immunology and Allergy, Department of Internal Medicine, Tri-Service General Hospital, National Defense Medical Center, Taipei, Taiwan; ^2^Division of Rheumatology, Immunology and Allergy, Department of Internal Medicine, Taichung Armed Forces General Hospital, Taichung, Taiwan; ^3^Graduate Institute of Medical Sciences, National Defense Medical Center, Taipei, Taiwan; ^4^Division of Cardiology, Department of Internal Medicine, Taichung Armed Forces General Hospital, Taichung, Taiwan; ^5^Department of Medical Education and Research, Taichung Armed Forces General Hospital, Taichung, Taiwan; ^6^Graduate Institute of Radiological Science, Central Taiwan University of Science and Technology, Taichung, Taiwan; ^7^Division of Cardiology, Department of Internal Medicine, Tri-Service General Hospital, National Defense Medical Center, Taipei, Taiwan

## Abstract

The diagnostic value and optimal cutoff level of cardiac troponin I in patients with sepsis have not been studied. In this single hospital retrospective study, we assessed the optimal cutoff value of troponin I for diagnosing non-ST-segment elevation myocardial infarction (NSTEMI) with type 1 myocardial infarction (MI) in patients with sepsis who had undergone a percutaneous coronary intervention from 2009 to 2019. In total, 5,341 patients (excluding patients with chronic kidney disease) were included, of whom 277 had sepsis or septic shock. Of the 123 patients with non-ST-segment elevation acute coronary syndrome (NSTE-ACS) and sepsis, 77 (62.6%) were diagnosed with NSTEMI with type 1 MI. The receiver-operating characteristic curve showed an area under the curve (AUC) of 0.705 for diagnosis of NSTEMI with type 1 MI with a troponin I cutoff of >300 ng/L (sensitivity: 68.4%, specificity: 70.2%, Youden index: 0.386). Multiple linear regression showed no significant predictors of NSTEMI with type 1 MI. Troponin level and the Global Registry of Acute Coronary Events (GRACE) scores were correlated (*R*^2^ = 0.0625, *p* = 0.032) and showed comparable predictive value for 6-month mortality (AUC: 0.637 and 0.611, respectively, *p* = 0.7651). The optimal troponin I cutoff to effectively diagnose NSTEMI with type 1 MI in patients with sepsis was 300 ng/L.

## 1. Introduction

Acute myocardial infarction (AMI), defined by evidence of myocardial injury with a clinical condition consistent with acute myocardial ischemia and elevation in the level of at least one cardiac troponin above the 99th percentile upper reference limit (URL) [1, 2], is a major cause of disability and death in the western world. On the basis of initial electrocardiograms (ECG), AMI can be clinically classified into two groups: ST-segment elevation myocardial infarction (STEMI) and non-ST-elevation acute coronary syndrome (NSTE-ACS). Moreover, the clinical condition of NSTE-ACS with pathological correlate with myocardial necrosis is defined as NSTEMI. [3]

There are five types of myocardial infarction (MI). Type 1 MI is defined as atherothrombotic myocardial injury, caused by either an ulceration or a plaque rupture. Most type 1 MIs occur in patients with STEMI, but some occur in patients with NSTEMI. Type 2 MI is characterized by myocardial necrosis caused by inequality of myocardial oxygen supply and demand due to factor other than coronary plaque instability. Type 3 MI is defined as an MI that caused death in the absence of cardiac biomarkers. Type 4 and 5 MIs are intervention-related MIs [3].

Elevated troponin I may complicate cardiac catheterization in patients with sepsis. Many patients with a primary diagnosis of sepsis receive troponin testing [4] and have a higher rate of catheterization [5]. There are several ways in which sepsis can complicate AMI diagnosis. First, several studies have shown that sepsis is associated with elevated cardiac troponin [6–8]. Second, ST-T wave changes are frequently observed in patients with sepsis [9]. Both cardiac troponin elevation and ST-T wave changes can occur through sepsis-induced myocardial dysfunction (SIMD)—also known as septic cardiomyopathy [10–12]. Third, dyspnea is a frequent symptom of sepsis and also a common symptom of AMI, which may confuse physicians during diagnosis [13, 14].

Elevation of cardiac troponin is a diagnostic cornerstone for AMI diagnosis. However, many non-AMI conditions, including renal dysfunction and sepsis, may also cause elevated cardiac troponin [2]. A newer method—high-sensitive cardiac troponin T (hs-cTn)—has been introduced into clinical practice since 2010 [15], and it is preferred for the diagnosis of AMI over the old method [3]. However, this assay has been approved in the United States and Taiwan since 2017 and 2020, respectively [16, 17]. The method of hs-cTn was not prevalent in Taiwan before 2020; therefore, we conducted a pilot study by using cardiac troponin for diagnosing NSTEMI with type 1 MI in NSTE-ACS patients with sepsis or septic shock.

## 2. Materials and Methods

### 2.1. Study Participants

**Figure 1:**
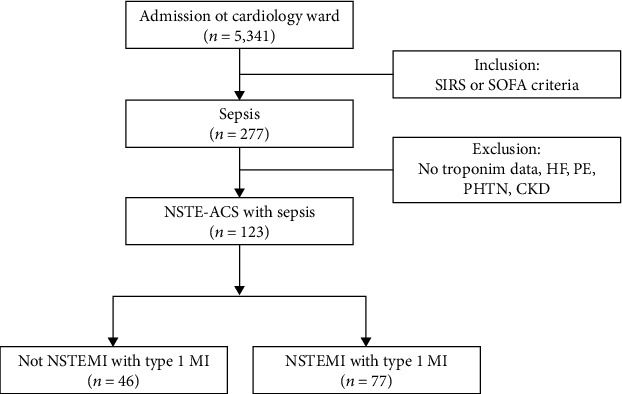
Flow diagram of the selection of study patients. CKD: chronic kidney disease; HF: heart failure; NST-ACS: non-ST-segment elevation acute coronary syndrome; NSTEMI: non-ST-segment elevation myocardial infarction; PE: pulmonary embolism; PHTN: pulmonary hypertension; SIRS: systemic inflammatory response syndrome; SOFA: Sequential Organ Failure Assessment.

We performed an observational and retrospective study in the Division of Cardiology at a secondary hospital in Taichung, Taiwan, between 2009 and 2019. The participant selection process is summarized in Figure 1. All adults (≥18 years) who were admitted to the cardiology ward during the study period were assessed for eligibility. Collected data included patients' demographics, troponin I, and creatinine levels, and data regarding underlying diseases such as hypertension (ICD-9-CM code: 401–405); chronic kidney disease (CKD; ICD-9-CM code: 585), defined as glomerular filtration rate < 60 mL/min/1.73 m^2^ for ≥3 months [18]; and diabetes mellitus (DM; ICD-9-CM code: 250). The study protocol was approved by the Institutional Review Board of Tri-Service General Hospital (study no. B202005110). The requirement for consent was waived because the study was retrospective.

### 2.2. Troponin I Assay

A paramagnetic particle, chemiluminescent immunoassay (AccuTnl Reagent Kit A78803, Beckman Coulter Inc., Brea, CA, USA) was used to measure cardiac troponin I. A cardiac troponin I level > 40 ng/L (99th percentile) was considered abnormal [19]. The lowest limit in our study was 10 ng/L, and the highest limit was 100,000 ng/L. If values > 100,000 ng/L, we used 100,000 ng/L for analysis. A single troponin I method was used during the study period. All patients received serial troponin tests except patients who required an immediate percutaneous coronary intervention (PCI) because of conditions such as cardiogenic shock, life-threatening arrhythmias, or chest pain despite medical management [3]. The measurement closest to the time of the PCI was used for the analysis.

### 2.3. Global Registry of Acute Coronary Event Score

The Global Registry of Acute Coronary Events (GRACE) score is used to predict in hospital deaths and 6-month mortality after discharge in patients with acute coronary syndrome. The GRACE scores (range 2–372) were calculated based on the patients' age, heart rate, systolic blood pressure, creatinine level, cardiac arrest at admission, ST-segment deviation on ECG, abnormal cardiac enzymes, and Killip class at admission before the PCI [20].

### 2.4. Clinical and Laboratory Data

We collected laboratory, clinical, and radiological data from the patients' medical records. Demographic data, vital signs, and medical history were also recorded. Blood samples were collected from all included patients within three days before the PCI. WBC counts, total bilirubin, and creatinine levels were measured. The date of death was also documented, and those <6 months were included for the analysis of prognosis.

### 2.5. Definition of Sepsis

Sepsis in our patients was defined according to the criteria for systemic inflammatory response syndrome, i.e., the occurrence of more than two of the following: leukocytosis (white blood cell, WBC > 12 × 10^9^/L) or leukopenia (WBC < 4 × 10^9^/L), hypothermia (<36.0°C) or fever (>38.0°C), and tachypnea (>20 breaths/min) or tachycardia (>90 beats/min) [21]. Patients who met the criteria for sequential organ failure assessment were also included [22]. Furthermore, patients with a suspected infection source who had been treated with one or more antibiotics were also included.

### 2.6. Exclusion Criteria

Patients with confounding factors that may impact the troponin level including chronic kidney disease (CKD), chronic heart failure, pulmonary embolism, or pulmonary hypertension [2] were excluded.

### 2.7. Definition of Non-ST-Segment Elevation Myocardial Infarction with Type 1 Myocardial Infarction

NSTE-ACS is defined as the occurrence of acute chest discomfort or symptoms of cardiac ischemia (e.g., shortness of breath or sweating) combined with significant ST-T wave changes (e.g., transient or persistent ST-segment depression, flat T waves, or T-wave inversion) [1, 2]. Patients with NSTE-ACS were suspected of having an NSTEMI and referred to our cardiologists for a PCI.

NSTEMI with type 1 MI was defined as patients referred for a PCI with imaging findings of embolization or decreased blood flow in a coronary artery caused by an intracoronary atherosclerotic lesion, such as ulceration, plaque rupture, erosion, or a fissure with thrombus [3].

### 2.8. Statistical Analysis

The Shapiro-Wilk test was used to assess the normality of distribution of continuous variables. Chi-squared tests were used to compare categorical variables. Continuous variables were reported as the median and interquartile range, or the mean ± standard deviation, depending on their distribution. Receiver-operating characteristic (ROC) curve analysis was used to determine the area under the curve (AUC) to evaluate the diagnostic ability of troponin I. The optimal cutoff value of troponin I for the NSTEMI with type 1 MI group was calculated with the Youden index (specificity − 1 + sensitivity) derived from the ROC analysis. ROC curves were compared to evaluate the predictive value of troponin I level and GRACE scores in 6-month mortality. Variables with a *p* value < 0.5 were considered significant in the univariate analysis and were included in a subsequent multivariate analysis. The results are reported as the *p* value and 95% confidence intervals (CI). All statistical analyses were conducted using the MedCalc Statistical Software version 20 (MedCalc Software Ltd., Ostend, Belgium; https://www.medcalc.org; 2020).

## 3. Results

A total of 5,341 patients were included in the study (Figure 1). In total, 3,770 patients did not satisfy the sepsis or septic shock criteria, and 277 patients experienced sepsis or fulfilled the septic shock criteria. After excluding patients with confounding factors, 123 patients with NSTE-ACS and sepsis/septic shock received a PCI, and 77 patients were diagnosed with NSTEMI with type 1 MI.

A total of 123 patients with NSTE-ACS and sepsis/septic shock received a PCI (Table 1). Their mean age was 78.95 ± 13.42 years in the NSTEMI with type 1 MI group and 73.95 ± 13.40 years in the no-NSTEMI group. In the NSTEMI with type 1 MI group, 48 patients were male, 31 had hypertension, 16 had DM, and the mean creatinine and troponin levels were 1.82 mg/dL and 6,128 ng/L, respectively. The GRACE score was 151.2 in the NSTEMI with type 1 MI group and 139.7 in the no NSTEMI group. Troponin I levels were not significantly different in the NSTEMI with type 1 MI group and no NSTEMI groups. Age and GRACE scores were significantly different in the NSTEMI with type 1 MI and no NSTEMI groups; however, sex, hypertension, DM, and creatinine levels were not significantly different.

**Figure 2:**
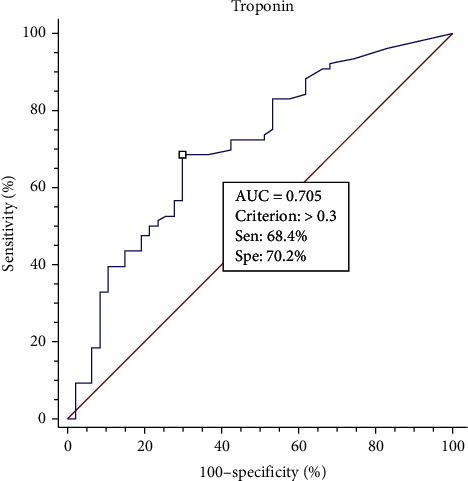
Receiver-operating characteristic curve of troponin I for the diagnosis of non-ST-segment elevation myocardial infarction with type 1 myocardial infarction in patients with sepsis. AUC: area under the curve; Sen.: sensitivity; Spe.: specificity.

The ROC curve (Figure 2) was used to calculate the optimal cutoff value of troponin as >300 ng/L in the NSTEMI with type 1 MI group. Our ROC curve had an AUC of 0.705 (*p* < 0.001). With a troponin I level cutoff of >300 ng/L, we could predict NSTEMI with type 1 MI with a sensitivity of 68.4% and specificity of 70.2%, and the Youden index was 0.386. The ROC curves for different combinations of predictors are shown in Figure S1. The results showed that troponin alone had the highest predictive value. The sensitivity and specificity of different troponin I level cutoff values are shown Table S1.

To examine whether the troponin values in our study were influenced by other factors, such as age, male sex, hypertension, DM, and creatinine levels, we conducted a univariable linear regression. A *p* value < 0.5 was defined as significantly different for the univariable linear regression. Hypertension, male sex, and age were significant factors and were included in the multiple linear regression model to predict troponin I. Multiple linear regression analysis (significance at *p* < 0.05) revealed no significant factors for troponin I in the NSTEMI with type 1 MI group (Table 2).

**Figure 3:**
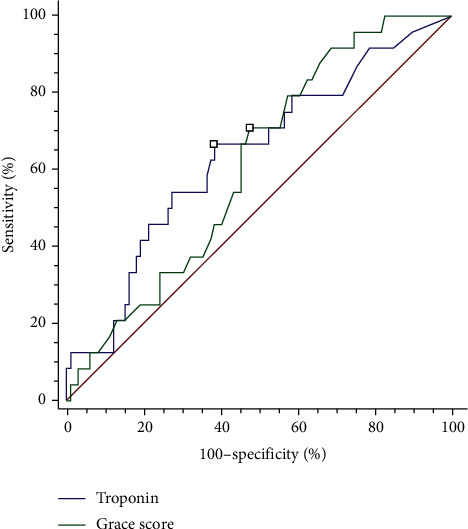
Comparison of the receiver-operating characteristic curves of troponin I and the Global Registry of Acute Coronary Events (GRACE) score for predicting 6-month mortality in patients with sepsis.

To examine the predictive value of troponin levels and GRACE scores in 6-month mortality, we compared the ROC curves. The ROC curves had AUCs of 0.637 and 0.611 for troponin and GRACE scores, respectively (*p* = 0.7651) (Figure 3). Both troponin level and GRACE scores could predict 6-month mortality and had comparable performance.

**Figure 4:**
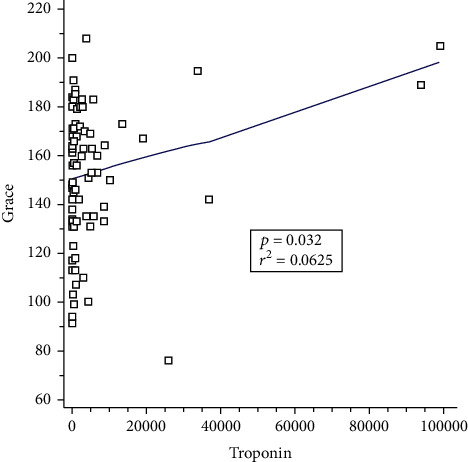
Correlation between the troponin I level and the Global Registry of Acute Coronary Events (GRACE) score in patients with sepsis.

Our study showed that the troponin I level directly correlated with the severity of myocardial injury. Moreover, troponin and GRACE scores could predict the 6-month outcome with similar predictive values. As expected, our findings revealed that troponin I levels correlated with GRACE scores. This correlation was validated by examining the troponin I levels and GRACE scores.  Figure 4 shows the correlation between troponin I levels and GRACE scores in the NSTEMI with type 1 MI group (*R*^2^ = 0.0625, *p* = 0.032).

## 4. Discussion

In total, 5,341 patients were included initially, and 123 patients with NSTE-ACS and sepsis or septic shock were included for analysis. Of these, 77 patients were diagnosed with NSTEMI with type 1 MI and 46 patients were not. Our study showed that a cutoff value of >300 ng/L for troponin I could predict NSTEMI with type 1 MI in patients with sepsis. The ROC curve had an AUC of 0.705 with a sensitivity of 68.4% and specificity of 70.2%. Factors such as age or creatinine level were not associated with troponin levels. Furthermore, both troponin levels and GRACE scores could predict 6-month mortality, and troponin levels were correlated with GRACE scores (*R*^2^ = 0.0625, *p* = 0.032). Our study is the first to report the optimal cutoff value of troponin I for NSTEMI with type 1 MI diagnosis in patients with sepsis and without CKD.

The ROC curves showed an AUC of 0.705. This may prove the utility of the troponin I level (>300 ng/L) for predicting NSTEMI with type 1 MI in patients with sepsis or septic shock. The specificity of our study is not high (70.2%). In clinical practice, clinicians may have performed a PCI in all the patients regardless of troponin I levels; however, NSTEMI with type 1 MI was diagnosed in only 62.60% (77/123) of the patients in our study. Thus, our approach—using a cutoff value of >300 ng/L—may increase diagnostic accuracy by approximately 8%. In addition, our study also had a high diagnostic rate compared to that in a previous study. In a retrospective study, Kim et al. [9] studied 397 patients with sepsis, of whom approximately 10% had CKD. In these patients, SIMD was diagnosed by transthoracic echocardiogram. The cutoff value of high-sensitive troponin I (hs-Tn I) was 668 ng/L with an AUC of 0.634 (sensitivity, 58.6%; specificity 59.1%). Although we used troponin I, which is considered a less sensitive marker for the diagnosis of NSTEMI [23], our study provided a higher diagnosis rate than the study by Kim et al. This may be because patients with CKD (a factor that may impact troponin levels) were excluded in our study.

Our study was similar to the aforementioned study. Since troponin is a specific biomarker for cardiac injury, other factors such as age, hypertension, DM, and male sex were not associated with it. Moreover, since we excluded patients with renal dysfunction, it is reasonable to assume that the creatinine levels were not related to troponin values in our study. The GRACE score is a well-known predictor of mortality in patients with AMI. It is a rapid method used worldwide to calculate cardiovascular risk in clinical assessment and guide patient triage and management [3]. In addition, troponin values may also predict mortality in patients with sepsis. Patients with sepsis admitted to intensive care units with elevated cardiac troponin I have been associated with a higher mortality rate [24–26]. Our study was similar to these studies; therefore, it is not surprising that troponin levels were found to correlate with the GRACE score in our study (Figure 4). Moreover, the troponin level had a similar predictive value as the GRACE score for 6-month mortality in patients with sepsis and NSTEMI with type 1 MI (Figure 3). We suggest that in practice, a simple troponin I cutoff value may be more practical for diagnosis than calculating GRACE scores.

The troponin complex is composed of troponin I, C, and T and is essential for cardiac muscle and skeletal contraction [27]. Cardiac troponin is exclusively of cardiac origin during fetal and embryonic development biomarker of myocardial injury [28]. However, a previous study showed a strong correlation between troponin T and troponin I levels in the diagnosis of AMI [8]. Sepsis may cause myocardial dysfunction and result in cardiac troponin elevation. SIMD is considered a syndrome that may present in many ways, including as myocardial abnormalities on echocardiography, myocardial damage with elevated cardiac biomarkers, and hemodynamic instability. SIMD is a frequent complication in patients with sepsis, with 40–60% of patients experiencing it. SIMD may involve both ventricles of the heart and may manifest as systolic/diastolic dysfunction [9]. The myocardial depression may be due to downregulation of beta-adrenergic receptors and the associated decrease in adrenergic response in cardiomyocytes [29, 30]. This hibernation-like phenomenon has been proposed to be due to oxidative stress-related inactivation of catecholamines [30].

Because of the high mortality rate (35.8%) among patients with sepsis with AMI [31], even a slight improvement in diagnostic accuracy for NSTEMI would be of considerable benefit for patients with sepsis. Taniel et al. included 2,602,854 patients with sepsis in the Healthcare Cost and Utilization Project National Inpatient Sample study in the United States from 2002 to 2011. Of these, 118,183 (4.5%) patients were diagnosed with AMI and most had NSTEMI. In clinical practice, most patients hospitalized with sepsis as the primary diagnosis concomitant with AMI receive conservative rather than invasive treatment (89.9% vs. 10.1%, respectively). Since patients who receive invasive treatment have a lower in-hospital mortality rate than those who receive conservative treatment, invasive risk stratification is recommended to determine patients with sepsis and AMI [31]. Although the mortality rate caused by AMI in patients with sepsis is very high, only a few patients receive appropriate vascular intervention clinically. Therefore, troponin I with a cutoff value of 300 ng/L may be used as a determinant for risk stratification in patients without CKD. This is a useful and straightforward metric and may encourage cardiologists to be more active in vascular intervention for patients with troponin levels beyond the cutoff value. However, more studies are needed to validate our findings. Furthermore, hs-Tn assays are increasingly being used in this field. Since hs-Tn is a more sensitive biomarker with a higher diagnostic performance than cardiac troponin [15], it may also be a promising candidate for which to evaluate cutoff values for NSTEMI with type 1 MI diagnosis in patients with sepsis.

The current study had several limitations. First, all patients were recruited before 2019. Therefore, our study is based on cardiac troponin I rather than hs-cTn, which is a newer method. Newer algorithms (0/1 or 0/2 hours; standard algorithms ≥ 3 hours) were not used for diagnosing NSTEMI with type 1 MI in our patients [3, 32]. However, our study had a more accurate prediction in diagnosing SIMD than another study that used hs-cTn [9]. We believe our pilot study may provide some additional information in studies using hs-cTn methods in the future. However, more studies on this aspect are needed. Second, it was a retrospective cohort study on prospectively collected data from a single secondary hospital in Taiwan. Therefore, our study had low statistical power for assessing the utility of the troponin cutoff value for diagnosing NSTEMI with type 1 MI, and more studies are needed. Third, our sample size was small, which may increase the margin of error and decrease the power of our study.

## 5. Conclusions

The datasets generated during and/or analyzed during the current study are not publicly available due to patient privacy and confidentiality, but are available from the corresponding author on reasonable request.

## Figures and Tables

**Table 1:** Baseline characteristics of patients with sepsis who received a percutaneous coronary intervention.

Characteristics	Total, *N* = 123	NSTEMI type 1, *N* = 77	No NSTEMI, *N* = 46	*p* value
Age (years)	77.08 ± 13.58	78.95 ± 13.42	73.95 ± 13.40	0.0481^∗^
Male sex, *n* (%)	71 (57.7%)	48 (62.3%)	23 (50.0%)	0.8759
Hypertension, *n* (%)	47 (38.2%)	31 (40.3%)	16 (34.8%)	0.0640
DM, *n* (%)	23 (18.7%)	16 (20.8%)	7 (15.2%)	0.1369
Creatinine (mg/dL)	1.78 ± 1.48	1.82 ± 1.34	1.72 ± 1.71	0.7151
GRACE score	146.9 ± 29.5	151.2 ± 29.3	139.7 ± 28.8	0.0362^∗^
Troponin (ng/mL)	5,065 ± 15,806	6,128 ± 16,356	3,286 ± 14,843	0.3368

^∗^ *p* < 0.05. DM: diabetes mellitus; GRACE: Global Registry of Acute Coronary Events; MI: myocardial infarction; NSTEMI: non-ST-segment elevation myocardial infarction; type 1: type 1 myocardial infarction.

**Table 2:** Regression analysis of factors associated with troponin I in patients with non-ST-segment elevation myocardial infarction with type 1 myocardial infarction.

Independent variable	Unadjusted				Adjusted			
	*R* ^2^	Coefficient	SE	*p*	*R* ^2^	Coefficient	SE	*p*
Age (years)	0.0283	0.2052	0.1387	0.1434^∗^	0.0212	0.1877	0.1395	0.1825
Male sex	0.0197	4.7106	3.8341	0.2231^∗^	···	4.7006	3.8776	0.2293
Hypertension	0.0116	−3.5699	3.8038	0.3510^∗^	···	−4.3869	3.8012	0.2522
DM	0.0040	2.5332	4.6154	0.5847	···	···	···	···
Creatinine	0.0008	−0.3573	1.4329	0.8038	···	···	···	···

^∗^ *p* < 0.5. SE: standard error of the coefficient; DM: diabetes mellitus; *R*^2^: coefficient of determination.

## Data Availability

The datasets generated during and/or analyzed during the current study are not publicly available due to patient privacy and confidentiality, but are available from the corresponding author on reasonable request.

## Supplementary Materials

Supplementary 1 Figure S1: receiver-operating characteristic curves of different combinations of predictors for the diagnosis of non-ST-segment elevation myocardial infarction with type 1 myocardial infarction in patients with sepsis. (A) Troponin, hypertension, and diabetes (AUC: 0.6580). (B) Troponin, age, creatinine, and the GRACE score (AUC: 0.6580). (C) Troponin, age, hypertension, diabetes, creatinine, male sex, and the GRACE score (AUC: 0.6157). AUC: area under the curve; GRACE: Global Registry of Acute Coronary Events.

Supplementary 2 Table S1: the sensitivity and specificity of different troponin cutoff values for diagnosing non-ST-segment elevation myocardial infarction with type 1 myocardial infarction in patients with sepsis.
