# Supplementary material for: Troponin I Cutoff for Non-ST-Segment Elevation Myocardial Infarction in Sepsis
Source: Mediators Inflamm. 2022 May 27;2022:5331474. doi: 10.1155/2022/5331474 (PMC9168824; doi:10.1155/2022/5331474)
Supplement: Supplementary 1 — Figure S1: receiver-operating characteristic curves of different combinations of predictors for the diagnosis of non-ST-segment elevation myocardial infarction with type 1 myocardial infarction in patients with sepsis. (A) Troponin, hypertension, and diabetes (AUC: 0.6580). (B) Troponin, age, creatinine, and the GRACE score (AUC: 0.6580). (C) Troponin, age, hypertension, diabetes, creatinine, male sex, and the GRACE score (AUC: 0.6157). AUC: area under the curve; GRACE: Global Registry of Acute Coronary Events. [file 5331474.f1.docx]

**Supplementary material**

|  |  |  |
| --- | --- | --- |
| (A) Troponin, hypertension, and diabetes. | (B) Troponin, age, creatinine, and the GRACE  score. | (C) Troponin, age, hypertension, diabetes,  creatinine, male sex, and the GRACE score. |

**Figure S1** Receiver-operating characteristic curves of different combinations of predictors for the diagnosis of non-ST-segment elevation myocardial infarction with type 1 myocardial infarction in patients with sepsis. (A) Troponin, hypertension, and diabetes (AUC: 0.6580). (B) Troponin, age, creatinine, and the GRACE score (AUC: 0.6580). (C) Troponin, age, hypertension, diabetes, creatinine, male sex, and the GRACE score (AUC: 0.6157).

AUC, area under the curve; GRACE, Global Registry of Acute Coronary Events
