# Supplementary material for: Troponin I Cutoff for Non-ST-Segment Elevation Myocardial Infarction in Sepsis
Source: Mediators Inflamm. 2022 May 27;2022:5331474. doi: 10.1155/2022/5331474 (PMC9168824; doi:10.1155/2022/5331474)
Supplement: Supplementary 2 — Table S1: the sensitivity and specificity of different troponin cutoff values for diagnosing non-ST-segment elevation myocardial infarction with type 1 myocardial infarction in patients with sepsis. [file 5331474.f2.docx]

**Table S1**. The sensitivity and specificity of different troponin cutoff values for diagnosing non-ST-segment elevation myocardial infarction with type 1 myocardial infarction in patients with sepsis.

| Troponin Cutoff (ng/L) | ≥ 10 | > 30 | > 60 | > 90 | > 200 | > 290 | > 300 | > 310 | > 650 | > 1,000 | > 3,100 | > 5,910 | > 10,160 | > 100,000 |
| --- | --- | --- | --- | --- | --- | --- | --- | --- | --- | --- | --- | --- | --- | --- |
| Sensitivity | 100 | 92.1 | 88.2 | 82.9 | 71.1 | 68.4 | 68.4 | 67.1 | 56.6 | 50 | 32.9 | 18.4 | 10.5 | 0 |
| Specificity | 0 | 31.9 | 38.3 | 46.8 | 57.4 | 68.1 | 70.2 | 70.2 | 72.3 | 78.7 | 91.5 | 93.6 | 93.6 | 100 |
